# Combining Pharmacological Countermeasures to Attenuate the Acute Radiation Syndrome—A Concise Review

**DOI:** 10.3390/molecules22050834

**Published:** 2017-05-19

**Authors:** Michal Hofer, Zuzana Hoferová, Daniel Depeš, Martin Falk

**Affiliations:** Department of Cell Biology and Radiobiology, Institute of Biophysics, v.v.i., Czech Academy of Sciences, Královopolská 135, 61265 Brno, Czech Republic; hoferovaz@centrum.cz (Z.H.); blackburn@ibp.cz (D.D.); falk@ibp.cz (M.F.)

**Keywords:** acute radiation syndrome, radioprotectors, radiomitigators, combined treatment, cytokines

## Abstract

The goal of combined pharmacological approaches in the treatment of the acute radiation syndrome (ARS) is to obtain an effective therapy producing a minimum of undesirable side effects. This review summarizes important data from studies evaluating the efficacy of combining radioprotective agents developed for administration prior to irradiation and therapeutic agents administered in a post-irradiation treatment regimen. Many of the evaluated results show additivity, or even synergism, of the combined treatments in comparison with the effects of the individual component administrations. It can be deduced from these findings that the research in which combined treatments with radioprotectors/radiomitigators are explored, tested, and evaluated is well-founded. The requirement for studies highly emphasizing the need to minimize undesirable side effects of the radioprotective/radiomitigating therapies is stressed.

## 1. Introduction

Although the search for suitable radiation countermeasures for radiation-associated injuries was initiated more than half a century ago, very few safe and effective radiation countermeasures for the most severe of these injuries, namely acute radiation syndrome (ARS), defined as ‘an acute illness caused by irradiation of the entire body (or most of the body) by a high dose of penetrating ionizing radiation in a very short period of time (usually a matter of minutes) [1], have been approved. This exception is represented by two granulocyte colony-stimulating factor (G-CSF)-based radiation countermeasures (Neupogen^®^ and Neulasta^®^) which have recently been approved by the United States Food and Drug administration (US FDA) for treatment of the hematopoietic ARS; both of these agents are radiomitigators for use after radiation exposure [1,2]. Not surprisingly both of the topics of “radioprotectors for use prior to exposure” and “therapeutic agents for post-exposure treatment” possess top priority among the research areas for radiological nuclear threat countermeasures [3].

It is well known that most of the agents tested for their abilities to protect from accidental radiation exposure or mitigate its health consequences, including the most effective ones, exhibit toxicities that can limit their usefulness. Therefore, various approaches have been used to decrease their undesirable side effects. One of the approaches is to combine two or more agents with the aim to reduce their toxicities while preserving, or even enhancing, the overall therapeutic outcome (e.g., [4,5]). This concise review deals with some of the most studied and promising experimental combinatory pharmacological interventions in ARS. The aim of the review is to emphasize the advantages of the approach of combined pharmacological treatment with radioprotectors/radiomitigators in patients with ARS.

## 2. Combinations Including Amifostine (WR-2721)

Amifostine (WR-2721), the most important representative of thiol radioprotectors, is a powerful radioprotective agent (a drug for use prior to irradiation); it acts on the principle of chemical radioprotection, i.e., predominantly by decreasing the levels of reactive oxygen species and hydrogen peroxide (e.g., [5]). Amifostine has been reported to reduce the effect of a radiation dose by a factor of up to 2.7 (the highest dose reduction factor (DRF) seen in a mouse 30-day survival model) [6]. However, due to its rather high toxicity, amifostine has not been approved for the treatment of ARS; nevertheless, it has found its use in radio- and/or chemotherapy-treated oncological patients (especially in head-and-neck cancer) as a selective protector of normal cells (e.g., [7,8]). Ongoing studies on amifostine using modern methods have confirmed its advantageous therapeutic qualities (e.g., [9]) and, therefore, the research on the combined use of amifostine with other agents in the treatment of ARS is worth mentioning.

Much work has been done on evaluation of combined effects of amifostine and antioxidative salts of various metals, like copper, zinc, or selenium (summarized by Weiss et al. [10]), both administered in the pre-irradiation regimen. It is noteworthy that the radioprotective efficacy of the metal-containing compounds themselves has been found to be low, whereas they have been found to significantly enhance the protective effects of amifostine and other thiol radioprotectors and reduce the thiol toxicity (e.g., [10,11]). Several hypotheses have been proposed explaining how metal salts positively influence the metabolism of amifostine; e.g., alkaline phosphatase activity in bone marrow cells has been shown to be significantly depressed after treatment with selenium, suggesting that a retardation of conversion of amifostine to its active free sulfhydryl form through the action of alkaline phosphatase might be responsible for the effects of selenium [10]; selenium has also been reported to induce glutathione peroxidase activity [12]. These findings and considerations have been reflected in later clinical attempts combining selenium and amifostine to attenuate undesirable effects of oncological radio/chemotherapy [13,14].

Another naturally-occurring antioxidant studied for the treatment of ARS in a combination with amifostine is vitamin E, a less effective radioprotector than synthetic thiols, like amifostine, but providing a longer window of protection against lethality (summarized by Weiss and Landauer [15]). In a 30-day survival experiment, WR-3689 (a thiol drug related to amifostine) and its combination with vitamin E (both administered pre-irradiation) have shown their DRFs of 1.35 and 1.49, respectively [16]. Later studies on rats’ whole bodies irradiated with a dose producing ARS have revealed mutually potentiating action of amifostine and vitamin E on radiation-damaged liver [17]. Recent findings have confirmed and extended the data from previous studies: an important observation has been obtained regarding the possibility to use low, non-toxic doses of amifostine and γ-tocotrienol, a vitamin E family member, for obtaining a high level of radioprotection [18].

In a pre-radiation administration setting, amifostine has also been experimentally combined with prostaglandin E_2_ (PGE_2_) or its synthetic analog, misoprostol. In a 30-day survival study, the DRF of amifostine alone, PGE_2_ alone, and amifostine + PGE_2_ in combination has been found to be 1.9, 1.45, and 2.15, respectively [19]. Prostaglandins have been found to stimulate erythroid and multilineage progenitor cells [20], as well as to enhance homing of hematopoietic stem cells through upregulation of the chemokine receptor CXCR4 and to stimulate hematopoietic stem cell survival by upregulation of apoptosis protein Survivin [21]. Both PGE_2_ and misoprostol have been reported to also be effective in the intestinal radiation syndrome [22]. Much later findings on the protective role of amifostine in preventing gastric damage produced by the prostaglandin synthesis inhibitor indomethacin have confirmed a positive influence of amifostine on the metabolism of prostaglandins [23].

Immunomodulators represent a wide group of substances stimulating the immune/hematopoietic system. Many of them, e.g., β-glucan and Broncho-Vaxom, shown here as examples of immunomodulators suitable for combined administration with amifostine for the treatment of ARS, possess no, or low, toxicity and are often commercially available. Their mechanism of action enables them to be administered in both protective and therapeutic regimens. Beta-glucan, a polyglucose, has been isolated from various sources. Thus, β-glucan from *Saccharomyces cerevisiae*, administered post-radiation, has been shown to significantly enhance the radioprotective effects of pre-irradiation-administered amifostine, their DRFs in a survival study being 1.37, 1.08, and 1.52 for amifostine alone, β-glucan alone, and amifostine + β-glucan, respectively [24]. The triple combination of pre-irradiation-administered amifostine, selenium, and β-glucan has been also reported as a very successful multiple-agent radioprotector [25]. In 2013, Pillai and Devi [26] followed up these studies with their experiments using β-glucan from the mushroom *Ganoderma lucidum*; they have reported a 100% mortality in untreated mice, but a 66% survival rate after 8 Gy exposure and post-irradiation administration of β-glucan, and even higher survival (83%) following the combination of pre-irradiation amifostine and post-irradiation β-glucan [26]. The authors stress that β-glucan is not toxic at the radioprotective dose [26]. For details on hematologic and radioprotective efficacy of β-glucan, see [27]. Broncho-Vaxom, a bacterial lysate, has been shown to potentiate in both protective and therapeutic administration settings the radioprotective effects of pre-irradiation amifostine [28,29]. The values of DRF in a survival study have been reported to be 1.92, 1.17, and 2.07 for amifostine alone, Broncho-Vaxom alone, and amifostine + Broncho-Vaxom, respectively, if both the drugs were applied pre-irradiation [29]. Recent studies on immunomodulators as components of drug combinations tested for their potential radioprotection/radiomitigation comprise polysaccharide from *Sipunculus nudus* and peptidoglycan. Polysaccharide from *Sipunculus nudus*, which is a species of unsegmented marine worms, administered post-irradiation jointly with interleukin-11 (IL-11) and G-CSF, has been reported to enhance the radioprotective action of pre-irradiation amifostine, as shown when parameters of hematopoietic, immune, and reproductive tissues in irradiated mice were evaluated [30]. Pre-irradiation amifostine and post-irradiation peptidoglycan, a polymer from the bacterial cell wall consisting of sugars and amino acids, have been found to synergistically promote the survival of irradiated mice through the amelioration of intestinal and bone marrow damage [31].

Granulocyte colony-stimulating factor (G-CSF) is a hematopoietic growth factor especially stimulating the production of neutrophils [32] and also showing antiapoptotic qualities [33]. For the detailed summary of its use in the treatment of ARS, see [34]. The combination of amifostine and G-CSF for the treatment of ARS has been aimed especially at evaluating the efficacy of the combined pre-irradiation amifostine and post-irradiation G-CSF therapy. In animal studies, this combination has been shown to be very effective [35,36], e.g., in a 30-day survival study, the LD50/30 values (radiation doses killing 50% of the experimental animals by day 30 after irradiation) for mice administered saline, G-CSF, amifostine, and amifostine + G-CSF have been reported to be 7.85 Gy, 8.30 Gy, 11.30 Gy, and 12.85 Gy, respectively [35]. In later publications dealing with the use of the amifostine + G-CSF combination in oncological patients, this approach has been thoroughly discussed in connection with both chemotherapy [37] and radiotherapy [38].

Metformin, a biguanide drug used in the treatment of type II diabetes, administered in a single dose 24 h after irradiation, has been found to significantly potentiate the radioprotective effects of amifostine in several cell types [39]. Metformin has been proposed to reduce endogenous reactive oxygen species and/or to slow down cell renewal progression and, as a result, to increase the time for repair; it has been suggested as a potentially useful agent for radiation countermeasures use [39].

**Figure 1:**
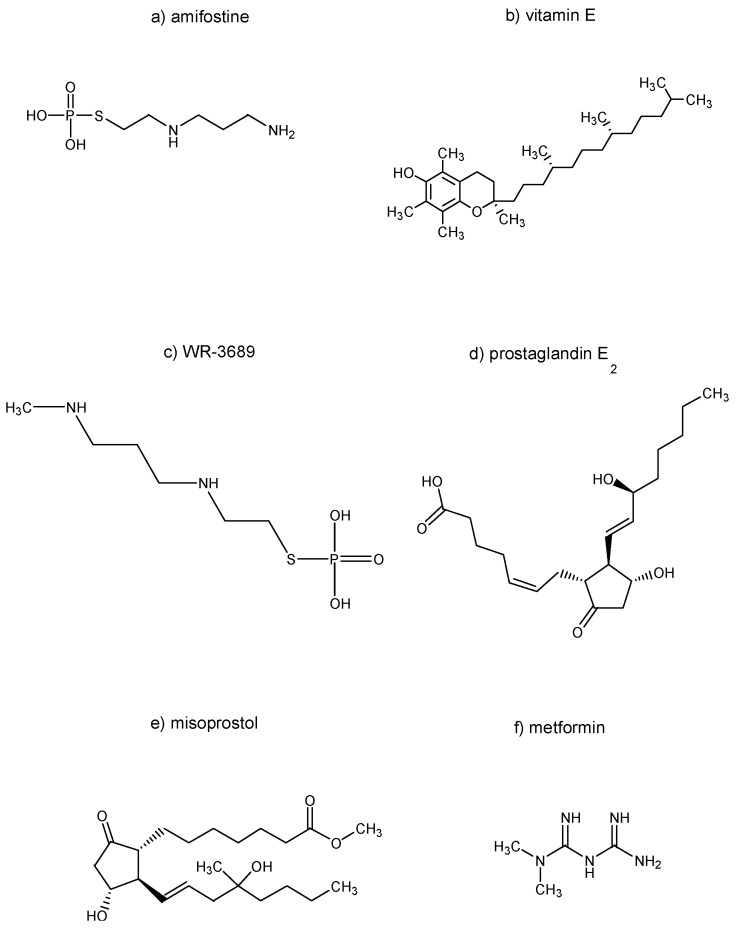
Chemical structures of selected substances explored in Section 2.

The chemical structures of the selected substances explored in Section 2, namely amifostine, vitamin E, WR-3689, prostaglandin E_2_, misoprostol, and metformin, are summarized in Figure 1.

## 3. Combinations without Amifostine or Other Thiol Radioprotectors

Whereas amifostine and related substances act in ARS mostly on the principle of chemical radiprotection (scavenging of free radicals), the combinations addressed in this paragraph include mostly agents functioning by influencing various metabolic and regulatory pathways in the mammalian organism. The latter agents have been often classified under the term “biological response modifiers” [40]. Their advantage is the possibility of both pre- and post-irradiation administration.

It has been found that the receptor action of extracellular adenosine acts radioprotectively in mice by the mechanisms of hypoxia induction due to the effects of the treatment on the cardiovascular system and enhanced regeneration of hematopoiesis; elevation of extracellular adenosine has been produced by combined administration of dipyridamole, a drug inhibiting the cellular uptake of adenosine, and adenosine monophosphate, an adenosine prodrug [41]. These findings and considerations have been confirmed in several studies and are summarized in a review [42]. Though not done on irradiated mice, the key for this topic were the results of a study describing the synergistic effects of combining dipyridamole + adenosine monophosphate with G-CSF on the production of neutrophils [43]; the effectiveness of this drug combination has been confirmed also under the conditions of ARS [44]. Later is was found that a selective stimulation of adenosine A_3_ receptors, e.g., by *N*^6^-(3-iodobenzyl)adenosine-5′-*N*-methyluronamide (IB-MECA), is responsible for the radioprotective and hematopoiesis-stimulating effects, previously observed after non-selective adenosine receptor activation, by the combination of dipyridamole and adenosine monophosphate [45]. Further experiments have shown that IB-MECA supports hematopoiesis-stimulating effects of G-CSF in sub-lethally irradiated mice when the two drugs are administered in a combination [46].

Cyclooxygenase inhibitors have been long studied for their ability to enhance hematopoiesis in an irradiated mammalian organism. With respect to their mechanism of action, they have been reported to remove, by inhibiting synthesis of PGE_2_, the negative feedback control of myelopoiesis played by PGE_2_ (for a detailed review, see [47]). Combined administration of diclofenac, a non-selective cyclooxygenase-1 and cyclooxygenase-2 inhibitor, and β-glucan, an immunomodulator, in single doses before irradiation of mice with a single sublethal dose of γ-rays, has been found to produce significantly better restoration of hematopoiesis in comparison with the mice administered with each of the drugs alone [48]. The same has become true when the two agents have been applied to mice before each of several repeated radiation doses [49]. When selective cyclooxygenase-2 inhibitors appeared, retaining the hematopoiesis-stimulating efficacy of non-selective cyclooxygenase inhibitors and producing less undesirable side effects, their representative, meloxicam, has been tested in a combined administration with IB-MECA, an adenosine A_3_ receptor agonist, in a post-irradiation regimen for the treatment of ARS. The combination of meloxicam + IB-MECA has been reported to be effective both when hematopoiesis was evaluated following a sub-lethal radiation dose [50] and when the survival of mice served as an outcome after a lethal radiation dose [51]. Meloxicam has also been found to stimulate endogenous production of G-CSF in irradiated mice [52] and to be able to substitute G-CSF in the treatment of ARS [53].

The abovementioned experimental combinatory ARS treatments including G-CSF represent only a part of the studies on the combined use of hematopoietic growth factors and cytokines in the treatment of the acute radiation disease. There exists a rather high number of papers on this topic already from the 1980s and 1980s. Some examples of these papers follow. As reported by Patchen et al., treatment of irradiated mice with the combination of β-glucan and G-CSF in a post-irradiation setting has been found to positively influence hematopoietic regeneration and survival of mice [54]. Enhanced radioprotection of mice has also been observed for the combinations of interleukin-1 (IL-1) and tumor necrosis factor [55] in a pre-irradiation therapeutic approach, IL-6 and G-CSF [56] administered after irradiation, or the triple combination of mast cell growth factor (C-kit ligand) + granulocyte-macrophage colony-stimulating factor (GM-CFC) + interleukin-3 (IL-3) also given post-irradiation [57]. Combination therapy of ARS using post-irradiation administration of G-CSF and synthokine SC-55494 (a synthetic high-affinity IL-3 ligand [58]) has been reported to be effective against post-irradiation neutropenia and thrombocytopenia in rhesus monkeys [59]. These experimental animals have also been used to evaluate the effects of the post-irradiation combinations of thrombopoietin (TPO), GM-CSF, and G-CSF; it has been stated that TPO significantly improves the performance of GM-CSF and G-CSF in alleviating severe neutropenia [60]. Significantly enhanced multilineage hematopoietic recovery in non-human primates has been observed following combined administration of megakaryocyte growth and development factor (MGDF) and G-CSF [61]. Intense attention has been paid to the topic of the post-irradiation administration of cytokine and growth factor combinations by the group of Hérodin and coworkers. Their experiments in mice have shown that the combinations of stem cell factor (SCF), Flt3-ligand, TPO, interleukin-3 (IL-3), and stromal-derived factor-1, administered 2 and 24 h after irradiation, significantly improved both short-term and long-term survival of the animals [62]. These findings have also been confirmed in subsequent studies on non-human primates where heavily irradiated (a whole-body dose of 7 Gy) monkeys were reported to be successfully treated from a severe bone marrow radiation syndrome when administered one-time combinations of SCF + glycosylated erythropoietin (EPO) + pegylated G-CSF [31] or pegylated G-CSF + SCF + Flt3-ligand + TPO + IL-3 [63] very early (2 h) after irradiation. The authors propose, as the main therapeutic mechanism of these cytokine cocktails, their anti-apoptotic action and emphasize the necessity of the early post-irradiation application of the drugs (“the sooner the better” [64]). Studies on the combined pharmaceutical modulation of ARS manifestation by pharmacological combinations comprising hematopoietic growth factors and cytokines still continue: a recent study has evaluated the efficacy of the joint administration of EPO, G-CSF, c-mpl receptor agonist romiplostim (a fusion protein analog of TPO), and nandrolone decanoate (a synthetic androgen) in mice following either a single post-irradiation dose within 2 h after irradiation, or five doses in five days following irradiation, as the most efficacious combination of EPO, G-CSF, and romiplostim that has been appraised [65].

Combinations of hematopoietic growth factors and cytokines have already found their use in the treatment of radiological accident victims. Various combinations of G-CSF, GM-CSF, EPO, SCF, and IL-3 have been administered to victims of accidents in Soreq (Israel, 1990), Neshviz (Belarus, 1992), Henan Province (China, 1999), Tokaimura (Japan, 1999), Prakan (Thailand, 2000), Fleurus (Belgium, 2006), and Dakar (Senegal, 2006) (summarized in [66]). Differing outcomes of the individual treatments are difficult to evaluate due to differing radiation sources, exposure doses, and other circumstances of the accidents.

Various natural antioxidants also play a role in radioprotective/radiomitigating combinations, e.g., mutual potentiation of the effects of post-irradiation administration of quercetin, an antioxidative flavonoid, and indralin, an adrenomimetic, in mice has been reported [67]. Enhanced hematopoietic protection has been obtained by combined pre-irradiation administration of the isoflavone genistein, an antioxidant and protein kinase inhibitor modulating signal transduction pathways, and captopril, an angiotensin-converting enzyme and vasodilator [68]. The combination of tocopherol succinate (a hemisuccinate ester of α-tocopherol belonging to the vitamin E family), an antioxidant, and AMD3100, an antagonist of chemokine receptor CXCR 4, enabling the displacement of hematopoietic stem cells and their subsequent migration to the peripheral blood, has been found to stimulate hematopoiesis in supralethally-irradiated mice [69]. Combined pre-irradiation administration of α-tocopherol acetate (an agent from the vitamin E family) and ascorbic acid (vitamin C), both antioxidants, has been reported to produce radioprotective properties, as shown by reduction of the numbers of chromosome aberrations in the bone marrow of rats [70]. A mixture of dietary antioxidants, including L-selenomethionine (an aminoacid), vitamin C, vitamin E succinate, α-lipoic acid (an organosulphur compound derived from octanoic acid), combined with *N*-acetyl-cysteine (an antioxidative drug and dietary supplement) has been found to protect hematopoietic cells and improve survival after its pre- or post-irradiation administration to X-irradiated mice [71] or post-irradiation administration to proton-irradiated mice [72].

Attention has also been paid to combinations of herbal extracts, e.g., Gupta and coworkers have shown potentiating effects of post-irradiation treatment with *Picrorrhiza kurroa* extract on pre-irradiation radioprotective efficacy of *Podophyllum hexandrum* extract [73]. Both the herbs are considered antioxidants, and *Podophyllum hexandrum* is also known for its anticancer effects.

An interesting report concerning radioprotective properties of the combination of selenium, zinc, and manganese with *Lachesis muta* (a pit viper species) venom in whole-body irradiated rats has been published by Crescenti and coworkers [74]. The authors call the combination an immunomodulator which positively affects radiosensitive tissues, including those of the gastrointestinal and hematopoietic systems [74].

Of additional interest are recent studies on pharmacological reduction of radiation-induced gastrointestinal toxicity by inhibitors of prolyl hydroxylase domain-containing enzymes (PHDs) whose administration has resulted in the stabilization of hypoxia-inducible factors (HIFs) protecting important cellular compartments from radiation-induced damage [75]. Combined administration of PHDs with glycogen synthase kinase-3 (GSK-3) inhibitors, decreasing the induction of p53-upregulated modulator of apoptosis (PUMA), has been proposed for radioprotection [76].

**Figure 2:**
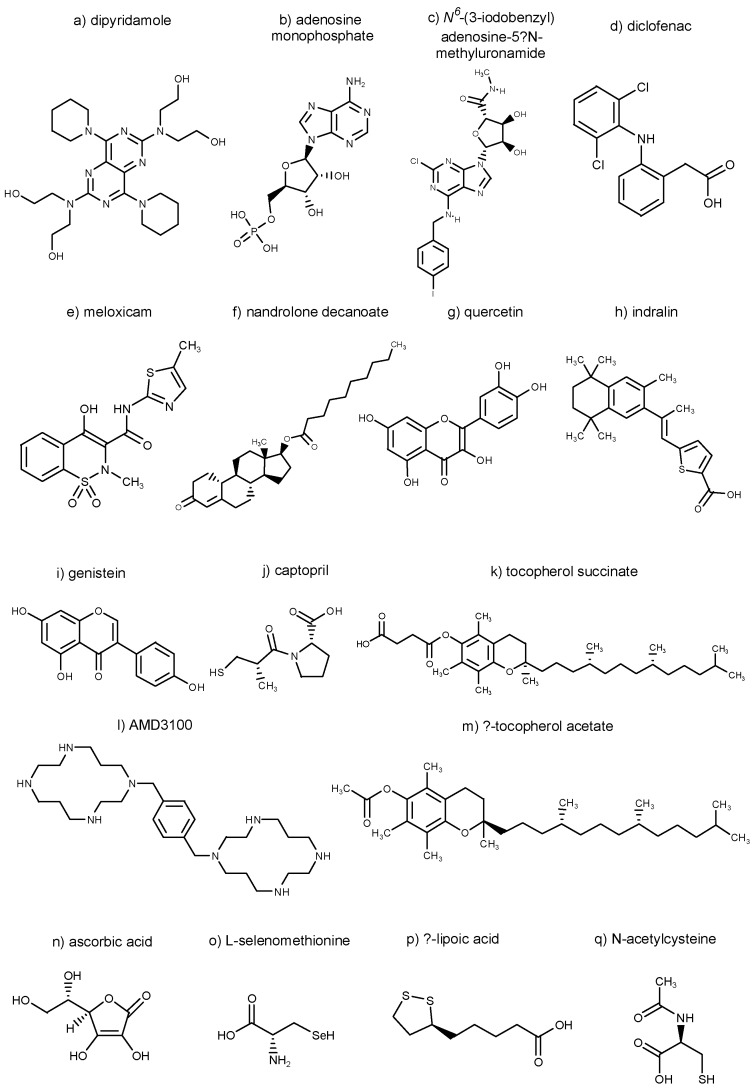
Chemical structures of selected substances explored in Section 3.

Chemical structures of selected substances explored in Section 3, namely dipyridamole, adenosine monophosphate, *N*^6^-(3-iodobenzyl)adenosine-5′-*N*-methyluronamide, diclofenac, meloxicam, nandrolone decanoate, quercetin, indralin, genistein, captopril, tocopherol succinate, AMD3100, α-tocopherol acetate, ascorbic acid, L-selenomethionine, α-lipoic acid, and *N*-acetylcysteine, are summarized in Figure 2.

Agents mentioned in this review that have been used in pharmacological combinations in attempts to modify the course of ARS are summarized in Table 1.

## 4. Discussion and Conclusions

As follows from the above summary of the findings on pharmacological modulation of ARS by combined drug treatment, this topic has required long-term attention. It can be deduced from the literature that, whereas in the years of the Cold War researchers focused their studies predominantly on the evaluation of “true radioprotectors”, i.e., chemical radioprotectors effective at pre-irradiation administration, current efforts are concentrated especially on compounds usable in therapeutic post-irradiation treatment approaches. Summaries of therapeutic principles for post-irradiation approaches to the treatment of ARS due to radiation accidents or contingent terrorist attacks can be found in several publications (e.g., [77,78]).

ARS is connected with multi-organ involvement, or even multi-organ failure [78]. Therefore, its general medical management is complex in essence and can comprise, e.g., administration of antibiotic, antimycotic, and antiviral substances (e.g., [79]), drugs for maintaining homeostasis through supporting renal function (e.g., [80]), transplantation of hematopoietic stem cells (e.g., [81]), or drugs modulating the course of the cutaneous radiation damage (e.g., [82]). These aspects of the treatment of the acute radiation disease exceed the thematic extent of this review and should be studied from other sources. This article has focused on pharmacological approaches directly influencing the radiation damage and those aimed preferentially at the hematopoietic radiation syndrome which represents the primary challenge in the case of whole-body acute exposure over 2 Gy [83].

As documented by the rich literature data, the aim of achieving a high efficacy of pharmacological reduction of the acute radiation damage in connection with a low toxicity of the therapy by means of combining suitable drugs has represented a challenge for a number of researchers over a long period. Many of the treatment schemes confirm the correctness of the idea; the values of important parameters after combined treatment are often additive or synergistic when compared to those following administration of the individual components of the combinations studied. According to the opinion of the authors of this review, the future of the research consists especially in studies on post-irradiation approaches which correspond more to the current dangers and demands in comparison with classical chemical radioprotectors set for pre-irradiation protective administration. Cytokines will undoubtedly remain the most efficacious component of the combined therapies of ARS. However, supplementation of the cytokine cocktails with some of the other drugs mentioned in this review would be worthy of study. Moreover, it should be taken into consideration that the most effective drug combination utilizing high doses of its individual components do not always represent the optimum general outcome. Development of effective combinations of radioprotectors/radiomitigators using doses of their components that are as low as possible, which would, thus, show the lowest possible intensities and incidences of their undesirable effects, might be the goal of some of the future studies on this topic. In this way, the aim of obtaining pharmacological approaches, which would be well tolerated in patients with ARS, could be achieved.

## Figures and Tables

**Table 1:** Summary of agents tested in pharmacological combinations in attempts to modify the course of the acute radiation syndrome (ARS).

Agent or Group of Agents	Predominant Radiomodifying Effect(s)	Reference Number(s)
Adenosine monophosphate	Stimulator of hematopoietic cell proliferation through adenosine receptor action	[41,42,43,44]
Alpha-lipoic acid	Antioxidant	[71,72]
AMD3100	Influences migration and homing of hematopoietic stem cells	[60]
Amifostine	Free radical scavenger	[5,6,7,8,9,10,11,12,13,14,15,16,17,18,19,20,21,22,23,24,25,26,27,28,29,30,31,32,33,34,35,36,37,38,39]
Ascorbic acid (vitamin C)	Antioxidant	[70,71,72]
Beta-glucan	Immunomodulator, stimulator of hematopoiesis	[24,25,26,27,54]
Broncho-Vaxom	Immunomodulator, stimulator of hematopoiesis	[28,29]
Captopril	Vasodilator	[68]
Diclofenac	Inhibitor of prostaglandin synthesis, stimulator of myelopoiesis	[47,48,49]
Dipyridamole	Enhances adenosine receptor action, stimulator of proliferation of hematopoietic cells	[41,42,43,44]
Erythropoietin (EPO)	Hematopoietic growth factor, stimulator of erythropoiesis	[31,65,66]
Flt3-ligand	Hematopoietic growth factor, stimulator of hematopoiesis	[62,63]
Genistein	Antioxidant	[68]
Glycogen synthase kinase-3 (GSK-3) inhibitor	Regulator of apoptosis	[76]
Granulocyte colony-stimulating factor (G-CSF)	Hematopoietic growth factor, stimulator of hematopoiesis	[30,31,32,33,34,35,36,37,38,52,53,54,56,58,61,63,65,66]
Granulocyte-macrophage colony-stimulating factor (GM-CSF)	Hematopoietic growth factor, stimulator of hematopoiesis	[57,60,66]
Indralin	Adrenomimetic	[67]
Inhibitors of prolyl hydroxylase domain-containing enzymes (PHDs)	Antioxidants	[75,76]
Interleukin-1 (IL-1)	Cytokine, regulator of immune response, inflammation, and hematopoiesis	[55]
Interleukin-3 (IL-3)	Cytokine, regulator of production of granulocytes and macrophages	[57,63,66]
Interleukin-6 (IL-6)	Cytokine, stimulator of myelopoiesis	[56]
Interleukin-11 (IL-11)	Cytokine, stimulator of hematopoiesis and lymphopoiesis	[30]
*Lachesis muta* venom	Immunomodulator (?)	[74]
Megakaryocyte growth and development factor (MGDF)	Hematopoietic growth factor, stimulator of thrombopoiesis	[61]
Meloxicam	Inhibitor of prostaglandin synthesis, stimulator of myelopoiesis	[50,51,52,53]
Metformin	Antioxidant, modulator of cell renewal	[39]
*N*^6^-(3-iodobenzyl)adenosine-5′-*N*-methyluronamide (IB-MECA)	Stimulator of hematopoietic cell proliferation through adenosine receptor action	[45,46,50,51]
*N*-acetyl-cysteine	Antioxidant	[71,72]
Nandrolone decanoate	Anabolic effects	[65]
Peptidoglycan	Immunomodulator, stimulator of hematopoiesis	[31]
Picrorhiza kuroa extract	Antioxidant	[73]
*Podophyllum hexandrum* extract	Antioxidant	[73]
Polysaccharide from Sipunculus nudus	Immunomodulator, stimulator of hematopoiesis	[30]
Prostaglandin E_2_ and prostaglandin family members (misoprotol)	Modulators of proliferation of hematopoietic cells, protectors of intestinal tissue	[19,20,21,22,23]
Quercetin	Antioxidant	[67]
Romiplostim	Hematopoietic growth factor, stimulator of hematopoiesis	[65]
Salts of various metals (copper, manganese, selenium, zinc)	Antioxidants	[10,11,12,13,14,71,72,74]
Stem cell factor (SCF) (c-kit ligand, mast cell growth factor)	Hematopoietic growth factor, stimulator of hematopoiesis	[31,57,62,63,66]
Stromal-derived factor-1 (SDF-1)	Chemokine, influences migration of hematopoietic cells	[62]
Synthokine SC-55494	Cytokine, stimulator of hematopoiesis	[58,59]
Thrombopoietin (TPO)	Hematopoietic growth factor, stimulator of thrombopoiesis	[60,62]
Tumor necrosis factor (TNF)	Modulator of inflammation	[55]
Vitamin E and its family members	Antioxidants	[15,16,17,18,69,70,71,72]
